# Development of a new segmentation algorithm for “precontemplation” stage in lifestyle change among high-risk populations for lifestyle-related diseases

**DOI:** 10.3389/fpubh.2026.1769487

**Published:** 2026-04-10

**Authors:** Kei Hirai, Wakana Tanabe, Koichiro Oka, Yoko Sato, Satoshi Sasaki, Atsushi Mizuno, Jun Fukuyoshi, Seiichiro Yamamoto

**Affiliations:** 1 Graduate School of Human Sciences, The University of Osaka, Suita, Japan; 2 Faculty of Sport Sciences, Waseda University, Tokorozawa, Japan; 3 Graduate School of Public Health, Shizuoka Graduate University of Public Health, Shizuoka, Japan; 4 Department of Social and Preventive Epidemiology, School of Public Health, University of Tokyo, Tokyo, Japan; 5 Department of Cardiovascular Medicine, St. Luke's International Hospital, Tokyo, Japan; 6 Cancer Scan, Co., Ltd., Tokyo, Japan

**Keywords:** behavioral economics, digital health communication, health behavior change, lifestyle improvement, precision medicine, precontemplation stage, segmentation algorithm, transtheoretical model

## Abstract

**Introduction:**

Specific health guidance programs provide motivational support to individuals in the precontemplation stage of the Transtheoretical Model (TTM) to encourage behavioral changes. However, this stage may encompass heterogeneous attitudes beyond simple lack of health interest. This study aimed to develop a novel segmentation classification incorporating behavioral economics concepts to better characterize the precontemplation population and validate targeted interventions.

**Methods:**

We conducted a two-phase study among Japanese adults aged 40–59 years eligible for specific health guidance. Phase 1 (*n* = 1,125) involved systematic questionnaire development based on behavioral economics literature and expert interviews, followed by factor analysis and k-means clustering to create a segmentation algorithm. Phase 2 (*n* = 4,900) validated targeted motivational messages for each identified segment through randomized message testing. Primary outcomes included changes in behavioral stage and health behavior intentions.

**Results:**

Seven distinct segments were identified: ‘High health awareness and practices' (18.7%), ‘High risk perception and busy' (12.0%), ‘High health threat' (21.1%), ‘High health anxiety' (11.8%), ‘Procrastination and improvement interest' (12.7%), ‘Busy and no future image' (19.2%), and ‘Procrastination and improvement resistance' (4.5%). A simplified 9-item algorithm achieved 50.7% classification accuracy (κ = 0.42, 95% CI: 0.39–0.45) compared to the original clustering. Message testing revealed significant segment effects (*F* = 7.48, *p* < 0.001, η*p*^2^ = 0.015). ‘High risk perception and busy' and ‘High health threat' segments showed greatest responsiveness to targeted motivational messages (Cohen's *d* = 0.28–0.35), while the ‘Procrastination and improvement resistance' segment showed consistent low responsiveness except to gain-framed messages focusing on specific actions.

**Discussion:**

This novel segmentation approach provides deeper understanding of the precontemplation stage by identifying distinct subgroups with unique behavioral economic characteristics including present bias and reference point orientations. Findings suggest that tailored interventions considering these behavioral economic factors may be more effective than traditional stage-based approaches for engaging resistant populations in health behavior change.

## 1 Introduction

The prevention and management of lifestyle-related diseases represent a fundamental challenge in contemporary global health, with mounting evidence that traditional one-size-fits-all approaches have limited effectiveness in promoting sustained behavior change (1, 2). The Transtheoretical Model (TTM) proposed by Prochaska and Velicer has been widely adopted as a framework for understanding readiness for health behavior change, categorizing individuals into distinct stages from precontemplation to maintenance (3). However, recent systematic reviews have raised significant questions about the effectiveness of TTM-based interventions, particularly for individuals in the precontemplation stage who show no intention to change within the next 6 months (4, 5).

A fundamental limitation of traditional stage-based approaches lies in their lack of explicit recognition regarding heterogeneity within stages. The precontemplation stage, characterized by lack of intention to change, may encompass diverse psychological states and decision-making patterns that extend far beyond simple “resistance to change” (6). This recognition has catalyzed growing interest in more personalized intervention approaches. Meta-analytic evidence demonstrates that messages tailored to individual characteristics are significantly more effective than non-tailored messages, with effect sizes ranging from small to moderate across various health behaviors (7). More recently, a comprehensive meta-analysis of 702 experimental tests confirmed that motivational message matching significantly enhances persuasion across attitudes, intentions, and behavior, further supporting the value of personalized approaches (8). The proliferation of digital communication technologies has made such personalization increasingly feasible and cost-effective, opening new possibilities for precision health communication (9).

Behavioral economics offers valuable theoretical insights for developing personalized health interventions by explaining how systematic cognitive biases influence health decisions (10, 11). Two concepts are particularly relevant for understanding resistance to health behavior change: present bias, which describes the tendency to disproportionately weight immediate costs and benefits over future ones, and reference points, which serve as anchors for evaluating potential gains and losses from behavior change (12, 13). These concepts provide a theoretical framework for understanding why individuals might resist health behavior changes despite understanding their importance and recognizing potential benefits.

Recent research has demonstrated the potential of combining behavioral economic insights with digital message delivery systems. Text messages incorporating loss aversion principles have shown promise in promoting medication adherence (14), while messages addressing present bias have effectively encouraged preventive health behaviors (15). However, these approaches often apply behavioral economic principles uniformly across populations, potentially missing opportunities for more nuanced targeting based on individual differences in cognitive biases and decision-making patterns. The emerging concept of “personalized nudging” further supports this direction by suggesting that tailoring choice architecture to individual psychological profiles has the potential to enhance the effectiveness of behavioral interventions (16, 17). Recent empirical work has demonstrated the potential of personalized digital nudges in health-related contexts, such as online food choices, where individually tailored interventions outperformed generic approaches (18). Particularly relevant to our framework, Tripp et al. (19) showed that tailoring cigarette warning messages based on individual differences in loss aversion and delay discounting rates significantly influenced perceived message effectiveness, providing evidence that behavioral economic individual differences can inform targeted health communication. However, these approaches have primarily focused on single behavioral economic dimensions, and comprehensive segmentation incorporating multiple cognitive biases and decision-making patterns remains underexplored.

The present study proposes that integrating behavioral economics concepts into audience segmentation could significantly enhance the effectiveness of targeted health messages, particularly for understanding and addressing various forms of resistance to health behavior change. Our research addresses this gap through two primary objectives: first, to develop a novel segmentation algorithm that identifies distinct subgroups within the precontemplation population, incorporating behavioral economic factors such as present bias and reference points; and second, to develop and validate the effectiveness of tailored motivational messages for each identified segment, leveraging digital delivery methods for precise targeting.

By pursuing these objectives, we aim to advance both theoretical understanding of health behavior change resistance and practical approaches to health communication. This work has significant implications for various health promotion contexts, from clinical settings to public health campaigns, where engaging resistant populations remains a critical challenge with substantial implications for population health outcomes.

## 2 Materials and methods

This study employed a sequential two-phase design: development of a segmentation algorithm (Phase 1) and validation of targeted motivational messages (Phase 2). The study protocol received approval from the Research Ethics Committee of the National Cancer Center (Phase 1; Approval No. 2021-368) and the Ethics Committee of the Graduate School of Human Sciences, The University of Osaka (Phase 2; Approval No. 22108).

### 2.1 Phase 1: development of segmentation algorithm

#### 2.1.1 Study design and participants

We conducted an internet-based cross-sectional survey using research monitors from NEO MARKETING INC., Japan. Sample size was determined through power analysis to detect medium effect sizes (Cohen's *d* = 0.5) with 80% power at α = 0.05, yielding a minimum required sample of 800 participants. We oversampled to 1,125 to account for potential data quality issues and ensure adequate representation across behavioral change stages.

Eligible participants were adults aged 40–59 years who met at least one of the following criteria: self-reported eligibility for specific health guidance under Japan's health insurance system, previous abnormal health checkup results through specific health examinations, or high BMI (≥30 kg/m^2^ for men, ≥28 kg/m^2^ for women based on Japanese obesity guidelines). At screening, participants were asked: “Are you thinking about improving your lifestyle habits such as exercise and diet?” with five response options: (1) “No intention to improve,” (2) “Intending to improve (within approximately 6 months),” (3) “Intending to improve soon (within approximately 1 month) and have started making small changes,” (4) “Already working on improvements (less than 6 months),” and (5) “Already working on improvements (6 months or more).” Only participants who selected option 1 (“No intention to improve”) or option 2 (“Intending to improve within approximately 6 months”) were included in this study.

#### 2.1.2 Questionnaire development and content

The questionnaire was developed through a systematic three-stage process: (1) comprehensive literature review of behavioral economic approaches to health behavior change, (2) semi-structured interviews with 15 individuals in the precontemplation stage to identify relevant psychological constructs, and (3) expert panel review by three behavioral economists and two health communication specialists. Detailed methodology is available in a Ministry of Health, Labor and Welfare report (20).

The final questionnaire assessed multiple domains including demographic characteristics, health behavior history, decision-making patterns, current life satisfaction, future orientation, health status perceptions, and specific health behaviors (physical activity, dietary habits, sleep patterns, health monitoring). Behavioral economic measures included present bias assessment through temporal discounting scenarios, risk preferences using validated scales, loss aversion through framing experiments, and social norm sensitivity measurements.

#### 2.1.3 Statistical analysis

We conducted exploratory factor analysis using principal component analysis with varimax rotation on conceptually related item groups. Kaiser-Meyer-Olkin (KMO) test and Bartlett's test of sphericity confirmed factorability (KMO > 0.8, *p* < 0.001 for all analyses). Factors with eigenvalues > 1.0 and factor loadings > 0.40 were retained. Missing data were minimal (< 3% for any variable) and handled using multiple imputation with chained equations for main analyses.

Cluster analysis employed k-means clustering with optimal cluster number determined through multiple criteria: silhouette analysis, gap statistics, and theoretical interpretability. We tested 2–10 cluster solutions, selecting the optimal number based on silhouette coefficient maximization (target > 0.4). Cluster stability was assessed through bootstrap resampling (*n* = 1,000).

A simplified classification algorithm was developed using Chi-squared Automatic Interaction Detection (CHAID) decision trees with 10-fold cross-validation. The 26-variable clustering solution served as the training target. Classification accuracy was evaluated using confusion matrices, sensitivity, specificity, and Cohen's kappa with 95% confidence intervals. All analyses were conducted using R version 4.3.0 with packages cluster, rpart, and caret.

### 2.2 Phase 2: message validation study

#### 2.2.1 Study design and participants

We conducted a randomized message testing study using research monitors from CROSS MARKETING INC., Japan. Eligible participants were adults aged 40–69 years meeting the same health risk criteria as Phase 1. After classifying participants into segments using the Phase 1 algorithm, we aimed to recruit 700 participants per segment for a total sample of 4,900 participants, providing adequate power (>80%) to detect small-to-medium effect sizes (*d* = 0.2–0.3) in message comparisons.

#### 2.2.2 Message development and testing

Segment-specific messages were developed through an iterative process involving behavioral economists, health communication experts, and public health practitioners. Each message incorporated elements targeting specific behavioral economic characteristics: temporal framing (immediate vs. future consequences), reference point manipulation (gain vs. loss framing), social norm information, and specific implementation suggestions. Messages were standardized to approximately 200 words each to control for length effects (Table 1).

**Table 1:** Segment-specific messages in Phase 2.

Segment no.	Segment name	Message
Segment 1	High health awareness and health measures	You appear to be actively practicing a healthy lifestyle through health monitoring, exercise, and dietary considerations. Your health condition seems good, and you probably haven't been sick much recently. To maintain a healthy and active life for a long time, and to achieve an even better physique that is more toned and resilient, why not consider regularly checking your health status through activity tracking and regular weight/blood pressure measurements? This data can help you find the best approach for your current physical condition. It might be worthwhile to listen to expert advice during your regular health checkups.
Segment 2	High risk perception and busy	You seem to be mindful of maintaining healthy behaviors like exercise, diet, and sleep, showing awareness of the risks of lifestyle diseases and cancer. However, in your busy life, it might be difficult to maintain these behaviors consistently. Try making small changes in your daily routine, such as eating three meals regularly, using stairs during transfers, and limiting smartphone use before sleep. By setting specific goals for physical activity, you can achieve a more toned body and improved work performance, leading to a healthier version of yourself.
Segment 3	Procrastination with interest in improvement	You might be worried about your future health and considering trying to live more healthily. However, to maintain your current health status without developing serious illnesses over the next 10 years, you need to build a body that's resistant to disease. Many people in similar situations have achieved results through planned approaches to diet and exercise with clear goals. Why not try improving your lifestyle habits by setting specific goals, such as adjusting your food portions or using a smartwatch or pedometer to encourage stair use? Additionally, consulting with your primary care physician or health nurses during checkups might help you find methods for becoming healthier that suit your lifestyle.
Segment 4	Procrastination with indifference and resistance	Have you ever thought about whether you'll be healthy 10 years from now? Although you might think you should do something about your health, you may feel you don't have time to address it right now. If you develop lifestyle-related diseases, you'll face time burdens from regular hospital visits and potentially hundreds of thousands of yen in medical costs if hospitalized. Try doing something for your health just for the month before your health checkup—you might notice some changes. While you might think it's difficult to become healthy through individual effort alone, at least maintain regular health checkups so you can consult with experts immediately if needed.
Segment 5	High health anxiety	You might be feeling anxious about your future health. Let's recall a time when you were in good physical condition. What kind of person would you be if you had continued those practices? Looking ahead 10 years, now is the perfect chance to start healthy lifestyle habits to maintain your current condition. Why not start by making small healthy changes to your daily life, such as choosing vegetable-rich options when eating out, switching to healthier drinks, using stairs when possible, and limiting smartphone use to get earlier sleep? Consulting with your primary care physician or health nurses during checkups might help you find health methods that suit you. Many people are already working on healthy living with expert guidance.
Segment 6	Busy with no future image	Have you ever thought about whether you'll be healthy 10 years from now? With your busy daily life, you might feel you don't have time to work on your health right now. If you develop lifestyle-related diseases, you'll face time burdens from regular hospital visits and potentially hundreds of thousands of yen in medical costs if hospitalized. Why not start by setting small goals like weight or daily step counts, and make small changes to your daily life such as choosing vegetable-rich options when eating out, switching to healthier drinks, using stairs when possible, and limiting smartphone use to get earlier sleep? Consulting with your primary care physician or health nurses during checkups might help you find better methods that suit your busy lifestyle.
Segment 7	High health threat	I believe you fully understand the need for health behaviors like moderate exercise, balanced diet, and quality sleep to reduce your risk of future lifestyle diseases, cancer, and dementia, and you're just starting to put these into practice. First, let's monitor what changes occur after maintaining these new health behaviors for a while. Additionally, consulting with your primary care physician or health nurses during checkups might help you find health measures that suit you better. By accumulating these healthy behaviors daily, you can achieve a healthier body than you have now.

#### 2.2.3 Randomization and outcome assessment

Participants within each segment were randomly assigned to review one of seven messages using the survey platform's built-in randomization function with stratification by age and gender. Primary outcomes included changes in TTM stage (measured on a 5-point scale) and intention to perform 15 specific health behaviors (measured on 7-point Likert scales). Secondary outcomes assessed message receptivity (5-point scale), perceived relevance (5-point scale), and behavioral specificity.

Statistical analysis employed mixed-effects ANOVA with segment and message type as fixed factors and participant as random factor. Effect sizes were calculated using partial eta-squared (η*p*^2^) and Cohen's *d* with 95% confidence intervals. *Post-hoc* comparisons used Bonferroni correction for multiple testing. All statistical tests were two-tailed with significance set at *p* < 0.05.

#### 2.2.4 Ethical considerations

All participants provided informed consent through the online platform before participation. The consent process included detailed information about study purpose, procedures, expected duration, voluntary participation, right to withdraw without penalty, potential risks and benefits, data confidentiality measures, and contact information for questions. Personal information was protected through data anonymization using participant ID codes, secure storage on password-protected servers with restricted access, and compliance with institutional data retention policies. No financial incentives were provided to avoid undue influence on participation decisions.

## 3 Results

### 3.1 Phase 1: development of segmentation algorithm

#### 3.1.1 Participant characteristics

Of 1,125 participants recruited, 771 (68.5%) were male with mean age 55.9 years (*SD* = 7.6, range 40–59). Initial TTM classification showed 27.6% (*n* = 311) with no intention to improve, 27.0% (*n* = 304) intending to improve within 6 months, 14.8% (*n* = 167) preparing to change, 15.2% (*n* = 171) in action phase, and 15.3% (*n* = 172) in maintenance phase. Participants reported diverse health conditions including hypertension (34.2%), diabetes (18.7%), and hyperlipidemia (28.9%).

#### 3.1.2 Variable extraction and segment identification

Factor analysis on questionnaire items yielded 26 key variables for segmentation with good to excellent reliability. From 14 risk perception items, we extracted three factors: major disease risk perception (α = 0.84), lifestyle disease risk perception (α = 0.79), and mental health loss risk perception (α = 0.76). Additional factors included decision-making ability (α = 0.81), disease fear (α = 0.88), and lifestyle improvement self-efficacy (α = 0.85).

**Figure 1:**
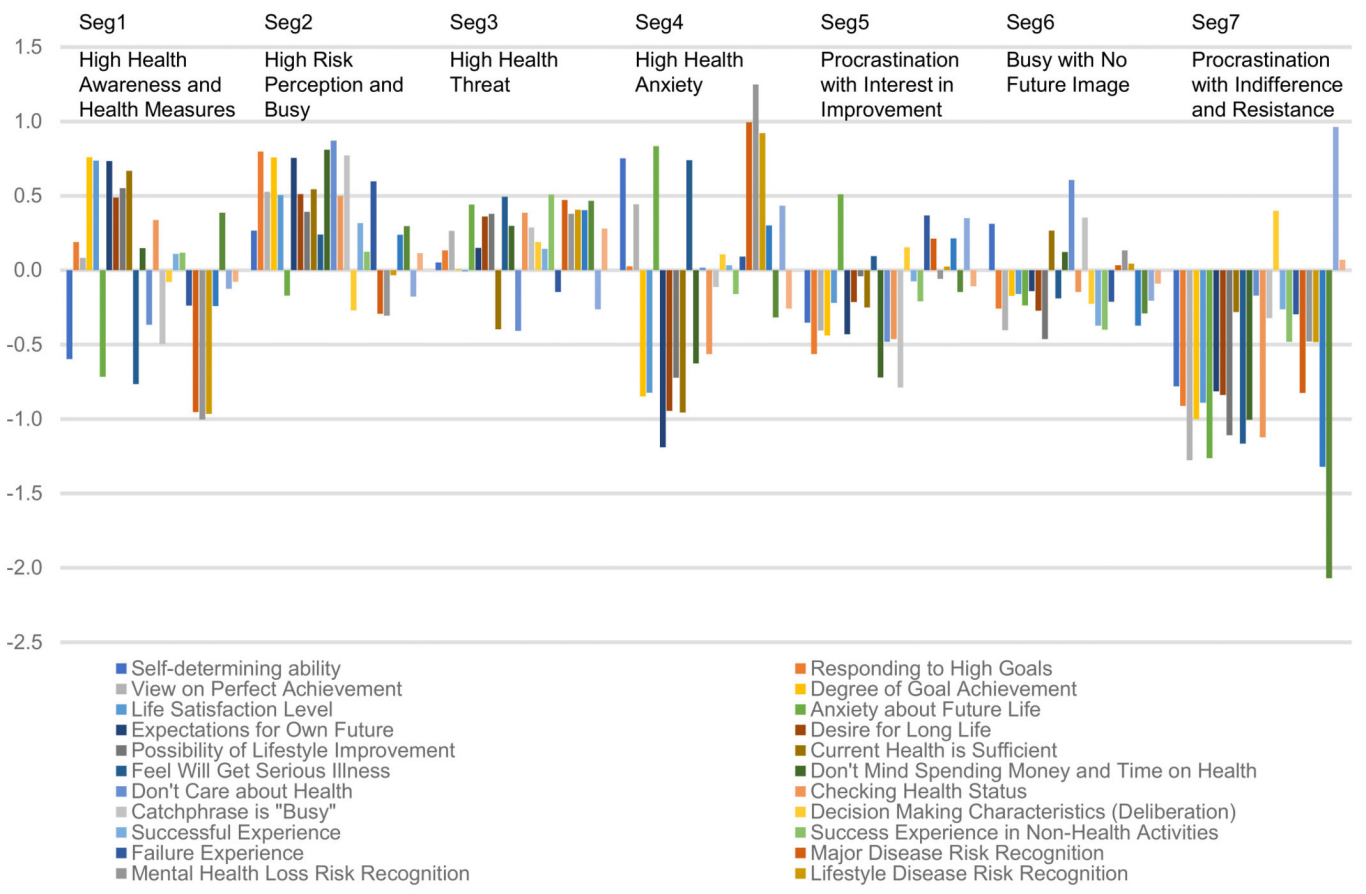
Standardized scores for 26 health variables across seven consumer segments.

K-means clustering revealed seven interpretable segments with acceptable internal consistency (average silhouette width = 0.42, range 0.35–0.51) (Table 2, Figure 1). The primary division emerged around future orientation (“Do you have expectations for your future?”), separating participants into goal-oriented vs. status quo reference points. Further segmentation occurred based on risk perceptions, health anxieties, and present bias tendencies, consistent with behavioral economics theory.

**Table 2:** Descriptive statistics of variables by cluster segment.

Variables	Seg1	Seg2	Seg3	Seg4	Seg5	Seg6	Seg7
	High health awareness and health measures	High risk perception and busy	High health threat	High health anxiety	Procrastination with interest in improvement	Busy with no future image	Procrastination with indifference and resistance
Self-determining ability	−0.597	0.266	0.053	0.751	−0.353	0.312	−0.780
Responding to high goals	0.190	0.798	0.133	0.027	−0.563	−0.258	−0.912
View on perfect achievement	0.083	0.527	0.264	0.443	−0.405	−0.403	−1.278
Degree of goal achievement	0.760	0.758	0.007	−0.847	−0.439	−0.173	−1.000
Life satisfaction level	0.737	0.504	−0.007	−0.823	−0.220	−0.161	−0.890
Anxiety about future life	−0.716	−0.171	0.442	0.835	0.511	−0.236	−1.263
Expectations for own future	0.734	0.754	0.150	−1.190	−0.430	−0.140	−0.814
Desire for long life	0.489	0.513	0.361	−0.945	−0.213	−0.271	−0.838
Possibility of lifestyle improvement	0.552	0.392	0.379	−0.723	−0.041	−0.463	−1.109
Current health is sufficient	0.669	0.544	−0.396	−0.956	−0.250	0.266	−0.282
Feel will get serious illness	−0.765	0.240	0.493	0.739	0.095	−0.190	−1.165
Don't mind spending money and time on health	0.148	0.810	0.299	−0.626	−0.721	0.123	−1.005
Don't care about health	−0.366	0.871	−0.407	0.017	−0.481	0.607	−0.171
Checking health status	0.339	0.500	0.386	−0.563	−0.463	−0.147	−1.122
Catchphrase is “Busy”	−0.497	0.772	0.287	−0.113	−0.788	0.353	−0.322
Decision making characteristics (Deliberation)	−0.080	−0.270	0.190	0.107	0.155	−0.225	0.399
Successful experience	0.110	0.317	0.144	0.034	−0.075	−0.373	−0.262
Success experience in non-health activities	0.117	0.124	0.509	−0.160	−0.209	−0.400	−0.481
Failure experience	−0.238	0.598	−0.146	0.092	0.368	−0.212	−0.296
Major disease risk recognition	−0.954	−0.293	0.472	0.994	0.213	0.034	−0.824
Mental health loss risk recognition	−1.004	−0.305	0.379	1.248	−0.057	0.133	−0.479
Lifestyle disease risk recognition	−0.966	−0.034	0.406	0.921	0.025	0.045	−0.482
Disease fear	−0.241	0.239	0.403	0.301	0.214	−0.373	−1.321
Lifestyle improvement self-efficacy	0.386	0.297	0.466	−0.318	−0.147	−0.291	−2.069
Present bias (Summer homework)	−0.125	−0.177	−0.263	0.433	0.350	−0.204	0.963
Risk avoidance level (Umbrella)	−0.078	0.115	0.280	−0.258	−0.109	−0.090	0.071

#### 3.1.3 Seven identified segments

1. High Health Awareness and Practices (*n* = 210, 18.7%): Characterized by high self-determination (*M* = 4.2, *SD* = 0.6), life satisfaction (*M* = 4.1, *SD* = 0.7), current health awareness (*M* = 4.3, *SD* = 0.5), and self-affirmation (*M* = 4.0, *SD* = 0.6), with low disease risk perception (*M* = 2.1, *SD* = 0.8). This group demonstrated proactive health engagement despite low perceived risk.

2. High Risk Perception and Busy (*n* = 135, 12.0%): Goal-oriented individuals (*M* = 4.0, *SD* = 0.7) with high life satisfaction (*M* = 3.9, *SD* = 0.6) but experiencing high work demands (*M* = 4.2, *SD* = 0.8) while maintaining capacity for health investment (*M* = 3.7, *SD* = 0.9).

3. High Health Threat (*n* = 237, 21.1%): Showed health improvement interest (*M* = 3.8, *SD* = 0.8) despite current dissatisfaction (*M* = 2.8, *SD* = 0.9), with moderate self-affirmation (*M* = 3.5, *SD* = 0.7) and risk awareness (*M* = 3.9, *SD* = 0.8).

4. High Health Anxiety (*n* = 133, 11.8%): Exhibited strong anxiety (*M* = 4.1, *SD* = 0.7), difficulty in self- determination (*M* = 2.6, *SD* = 0.9), limited longevity desire (*M* = 2.4, *SD* = 1.1), and high risk perception (*M* = 4.2, *SD* = 0.6) with procrastination tendencies (*M* = 3.7, *SD* = 0.8).

5. Procrastination and Improvement Interest (*n* = 143, 12.7%): Demonstrated self-determination ability (*M* = 3.6, *SD* = 0.8) but high future anxiety (*M* = 3.9, *SD* = 0.7), reluctance to invest in health (*M* = 2.9, *SD* = 0.9), and history of failed attempts (*M* = 3.8, *SD* = 0.9).

6. Busy and No Future Image (*n* = 216, 19.2%): Had difficulty with self-determination (*M* = 2.8, SD = 0.9), moderate current health perception (*M* = 3.2, *SD* = 0.8), reported being busy (*M* = 4.0, *SD* = 0.7), and showed strong procrastination tendencies (*M* = 4.1, *SD* = 0.6).

7. Procrastination and Improvement Resistance (*n* = 51, 4.5%): Exhibited strongest procrastination (*M* = 4.5, *SD* = 0.5), weakest risk perception (*M* = 1.9, *SD* = 0.7), current dissatisfaction without anxiety (*M* = 2.2, *SD* = 0.8), and reluctance to invest in health (*M* = 1.8, *SD* = 0.9).

#### 3.1.4 Simplified classification algorithm

**Figure 2:**
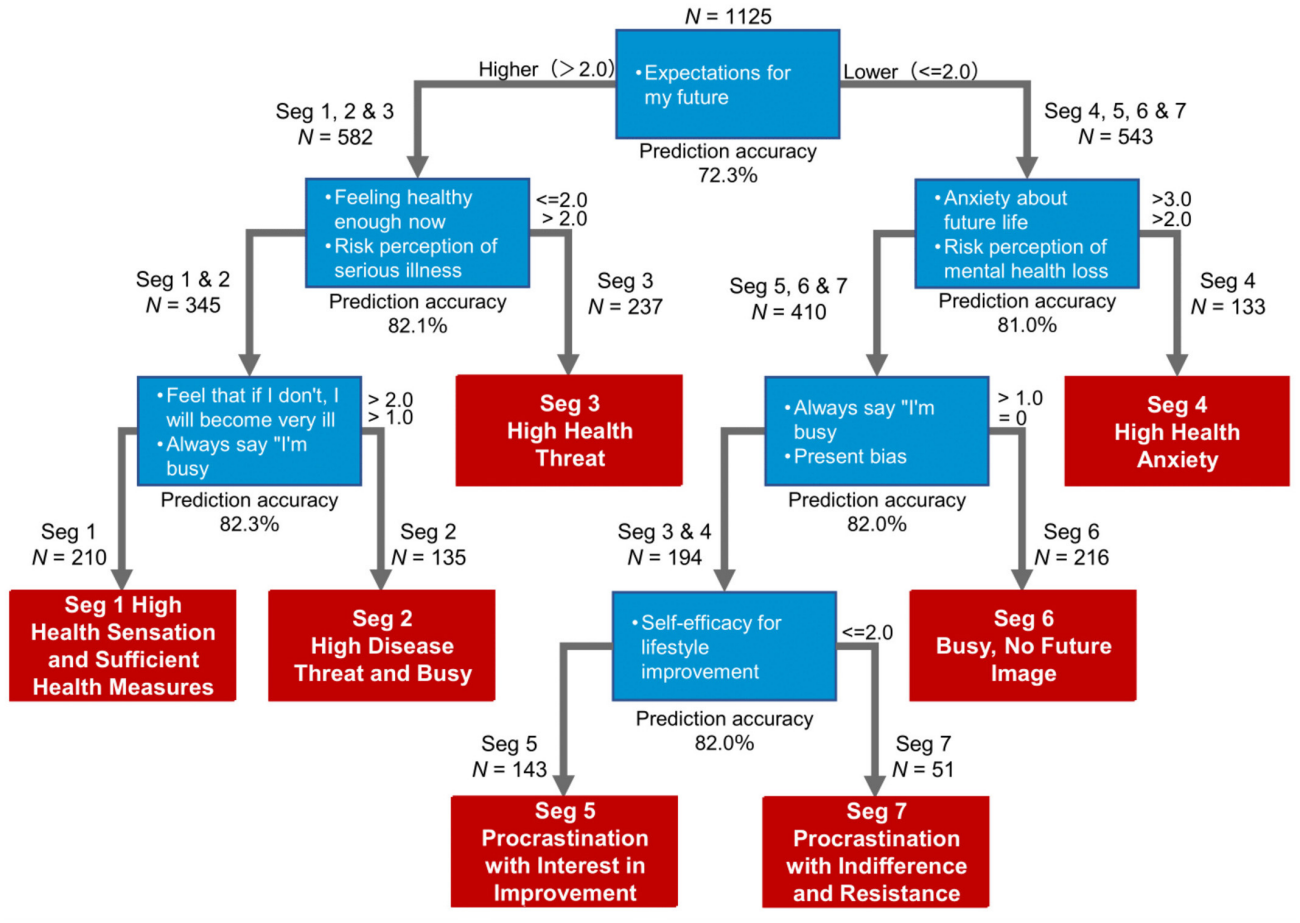
CHAID analysis results: simplified classification algorithm. *N* is the observed value in unsupervised machine learning.

CHAID analysis produced a simplified 9-item algorithm incorporating future expectations, current health perception, serious illness risk perception, future life anxiety, depression worry, current health status perception, busyness sense, lifestyle improvement efficacy, and childhood habits (Figure 2). This algorithm achieved 50.7% classification accuracy (κ = 0.42, 95% CI: 0.39–0.45) compared to the original 26-variable clustering, significantly above chance level for a 7-category classification (χ^2^ = 2,847.6, *p* < 0.001). It should be noted that classification errors were not uniformly distributed (Supplementary Tables S3, S4); confusion matrix analysis revealed that misclassifications most frequently occurred between segments sharing similar behavioral economic profiles (e.g., between Segments 1 and 2, or between Segments 5 and 7), suggesting that even misclassified individuals often received messages aligned with partially overlapping psychological characteristics.

### 3.2 Phase 2: message testing results

#### 3.2.1 Participant characteristics

In Phase 2, 4,900 participants were recruited (75.0% male; mean age 55.9 years, *SD* = 7.6) (Table 3). The target enrollment of 700 participants per segment was successfully achieved, with each of the seven segments comprising exactly 700 participants (total *n* = 4,900). This ensured adequate statistical power (>80%) to detect small-to-medium effect sizes (*d* = 0.2–0.3) for within-segment message comparisons across all segments. Demographic characteristics and health status indicators were comparable across segments (all *p* > 0.05 for age, gender, BMI, and health conditions), confirming successful randomization.

**Table 3:** Demographic and physical characteristics by cluster segment.

Characteristics	Segment 1		Segment 2		Segment 3		Segment 4		Segment 5		Segment 6		Segment 7		Total		*χ2*	*p*
	High health awareness and practices		High risk perception and busy		Procrastination and improvement interest		Procrastination and improvement resistance		High health anxiety		Busy and no future image		High health threat					
	*N*	%	*N*	%	*N*	%	*N*	%	*N*	%	*N*	%	*N*	%	*N*	%		
Sex (Male)	515	73.60	508	72.60	549	78.40	584	83.40	478	68.29	509	72.71	533	76.14	3,676	75.00	53.17	< 0.001
Experience of health checkups	389	55.60	416	59.40	352	50.30	299	42.70	341	48.70	363	51.90	402	57.40	2,562	52.30	56.85	< 0.001
Presence of abnormal findings in health checkups	280	40.00	335	47.90	358	51.10	303	43.30	336	48.00	316	45.10	400	57.10	2,328	47.50	94.05	< 0.001
History of treatment for chronic diseases	30	04.30	59	08.40	35	05.00	48	06.90	49	07.00	40	05.70	70	10.00	331	06.80	01.30	0.254
	Mean	SD	Mean	SD	Mean	SD	Mean	SD	Mean	SD	Mean	SD	Mean	SD	Mean	SD	F	p
Age	56.56	07.66	54.39	07.46	57.91	07.66	56.97	07.56	54.27	07.29	55.94	07.41	55.51	07.55	55.94	07.61	22.10	< 0.001
Height (cm)	167.03	08.05	167.61	08.42	167.27	07.85	167.97	08.42	165.94	08.03	166.92	08.42	168.00	08.03	167.25	08.20	05.38	< 0.001
Weight (kg)	64.87	12.67	67.65	12.91	67.37	13.49	71.59	16.91	67.88	15.55	67.81	14.37	68.44	13.28	67.94	14.35	13.47	< 0.001
BMI	23.14	03.60	23.97	03.63	23.97	03.94	25.44	08.36	24.50	04.72	24.21	04.20	24.15	03.89	24.20	04.92	14.07	< 0.001

#### 3.2.2 Message effects on behavioral stage

**Figure 3:**
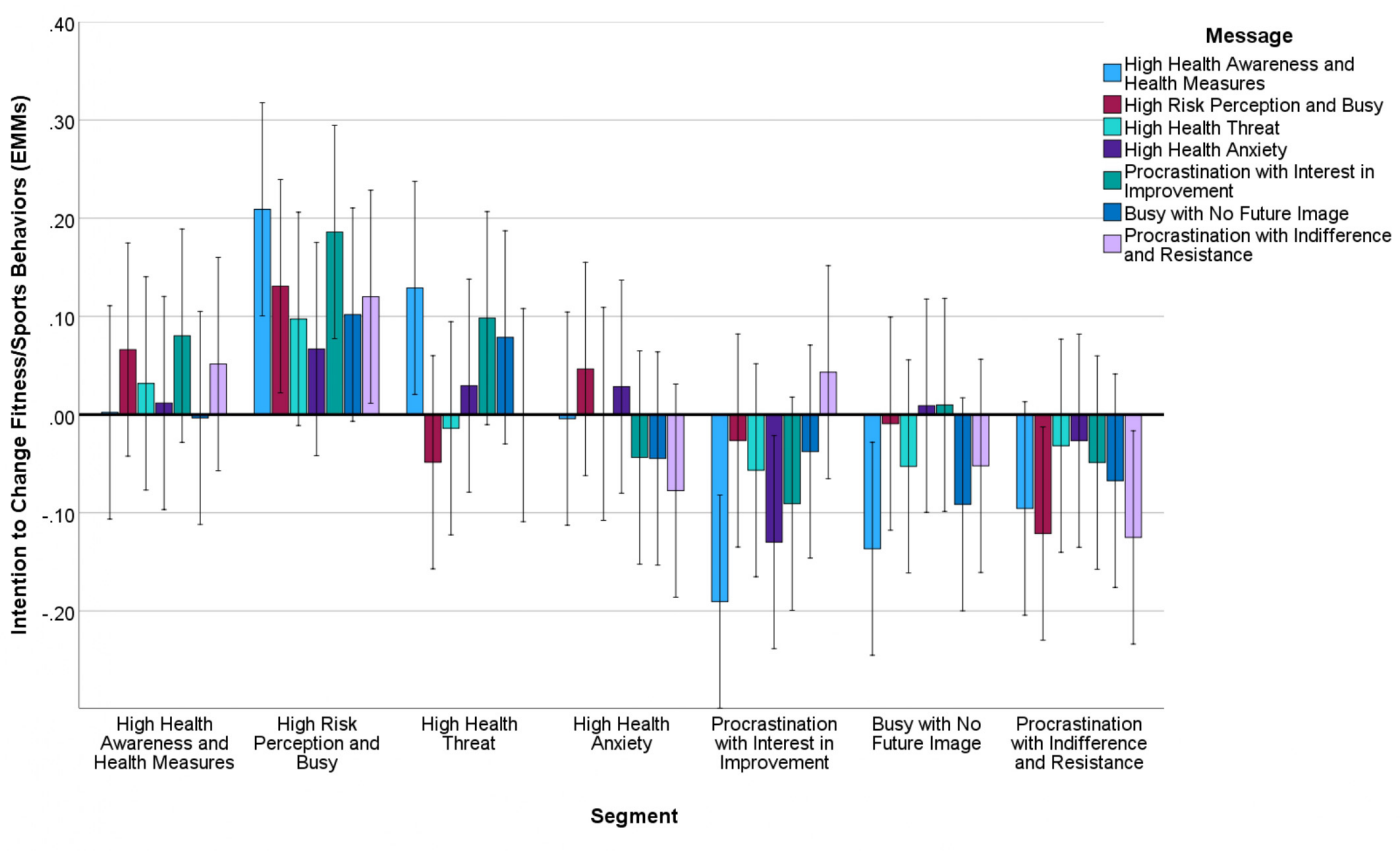
Estimated marginal means of stages of lifestyle improvement behavior after message presentation by segment. Covariates in this model are evaluated at stages of lifestyle improvement behavior (pre-intervention) = 2.73. Error bars: 95% CI.

Mixed-effects ANOVA revealed significant main effects for segment (*F* = 7.48, *df* = 6.4886, *p* < 0.001, η*p*^2^ = 0.015) and baseline stage (*F* = 4,606.49, *df* = 1,4886, *p* < 0.001, η*p*^2^ = 0.652; Table 4, Figure 3). The main effect of message type was not significant (*F* = 1.53, *df* = 6.4886, *p* = 0.17, η*p*^2^ = 0.002), but planned comparisons revealed significant segment-specific effects:

- High Risk Perception and Busy segment: Targeted message showed higher effectiveness than High Health Awareness message (mean difference = 0.31, 95% CI: 0.08–0.54, *p* < 0.05, *d* = 0.28).
- High Health Threat segment: High Health Awareness and Busy with No Future Image messages demonstrated greater effectiveness (mean differences = 0.26–0.31, 95% CI: 0.05–0.52, *p* < 0.05, *d* = 0.24–0.35).
- Procrastination and Improvement Resistance segment: Showed consistent low responsiveness across most messages, except for gain-framed action-specific messages (*d* = 0.41, 95% CI: 0.12– 0.70).

**Table 4:** Segment-specific message effects: ANOVA results.

Outcome variable	Main effect of segment		Main effect of message		Interaction		Covariate	
	*F*	*p*	*F*	*p*	*F*	*p*	*F*	*p*
Stages of lifestyle improvement behavior	7.48	< 0.001	1.53	0.17	0.99	0.48	4,606.49	< 0.001
Intention to change 15 types of health-related behaviors	12.54	< 0.001	1.10	0.36	0.60	0.97	8,221.86	< 0.001
Intention to change health management behaviors	12.56	< 0.001	0.96	0.45	0.63	0.96	7,660.90	< 0.001
Intention to change moderate physical activity	10.63	< 0.001	1.46	0.19	0.58	0.98	7,271.52	< 0.001
Intention to change fitness and sports-related behaviors	11.83	< 0.001	0.40	0.88	1.03	0.42	5,390.52	< 0.001

#### 3.2.3 Health behavior intention changes

**Figure 4:**
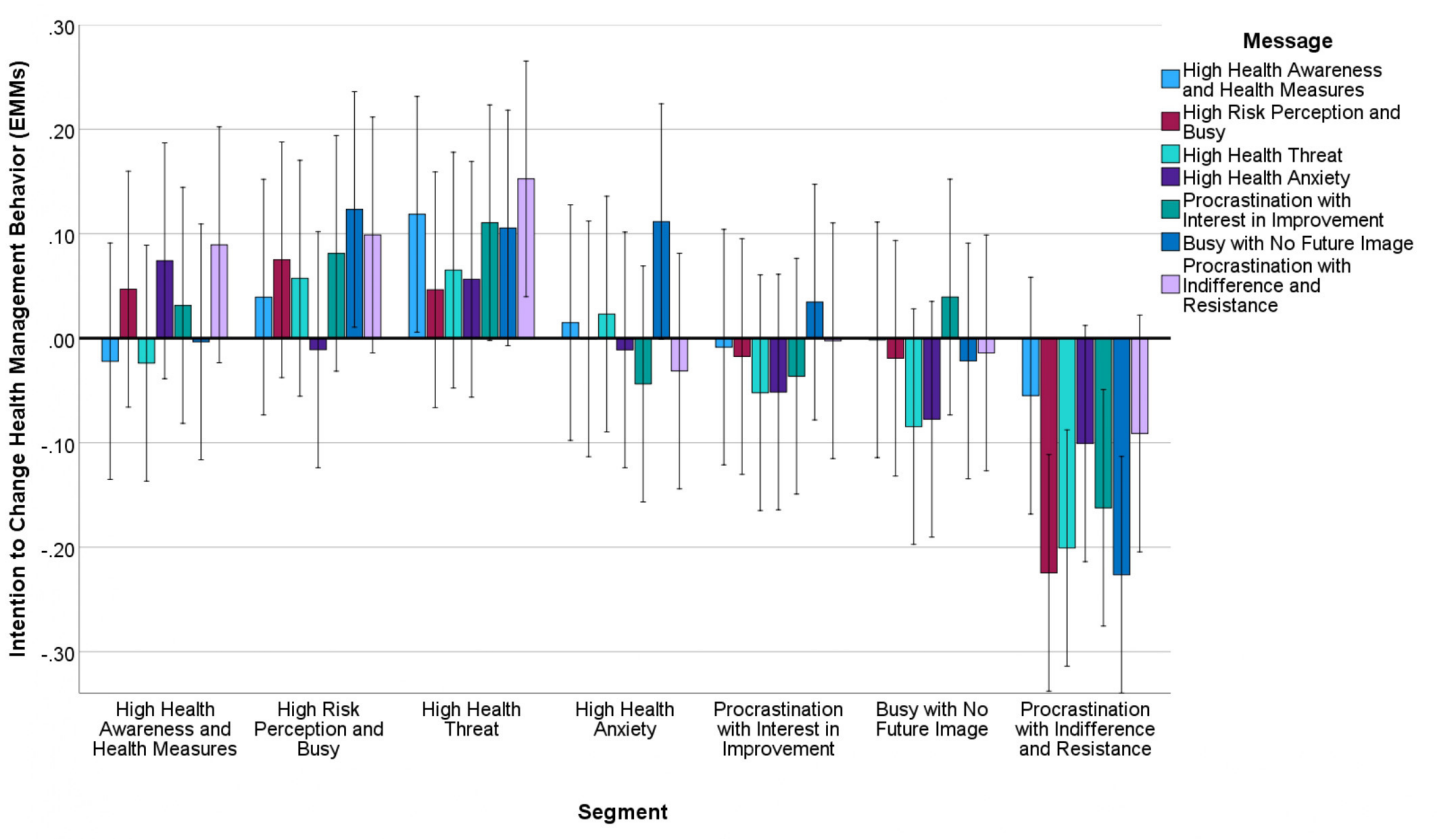
Estimated marginal means of intention to change health management behaviors after message presentation by segment. Covariates in this model are evaluated at intention to change health management behaviors (pre-intervention) = 0.000. Error bar: 95% CI.

**Figure 5:**
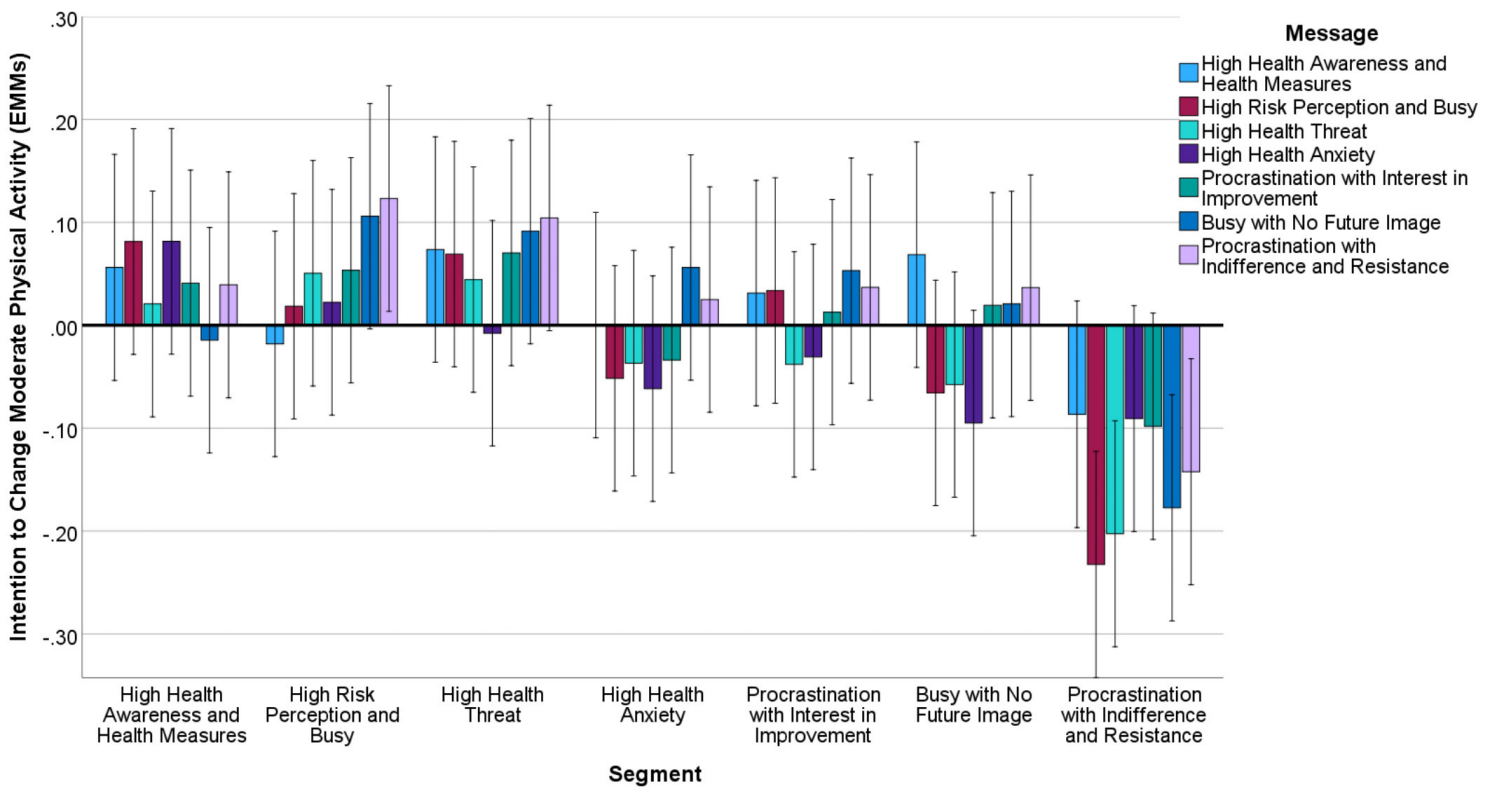
Estimated marginal means of intention to change moderate physical activity after message presentation by segment. Covariates in this model are evaluated at intention to change moderate physical activity (pre-intervention) = 0.000. Error bar: 95% CI.

**Figure 6:**
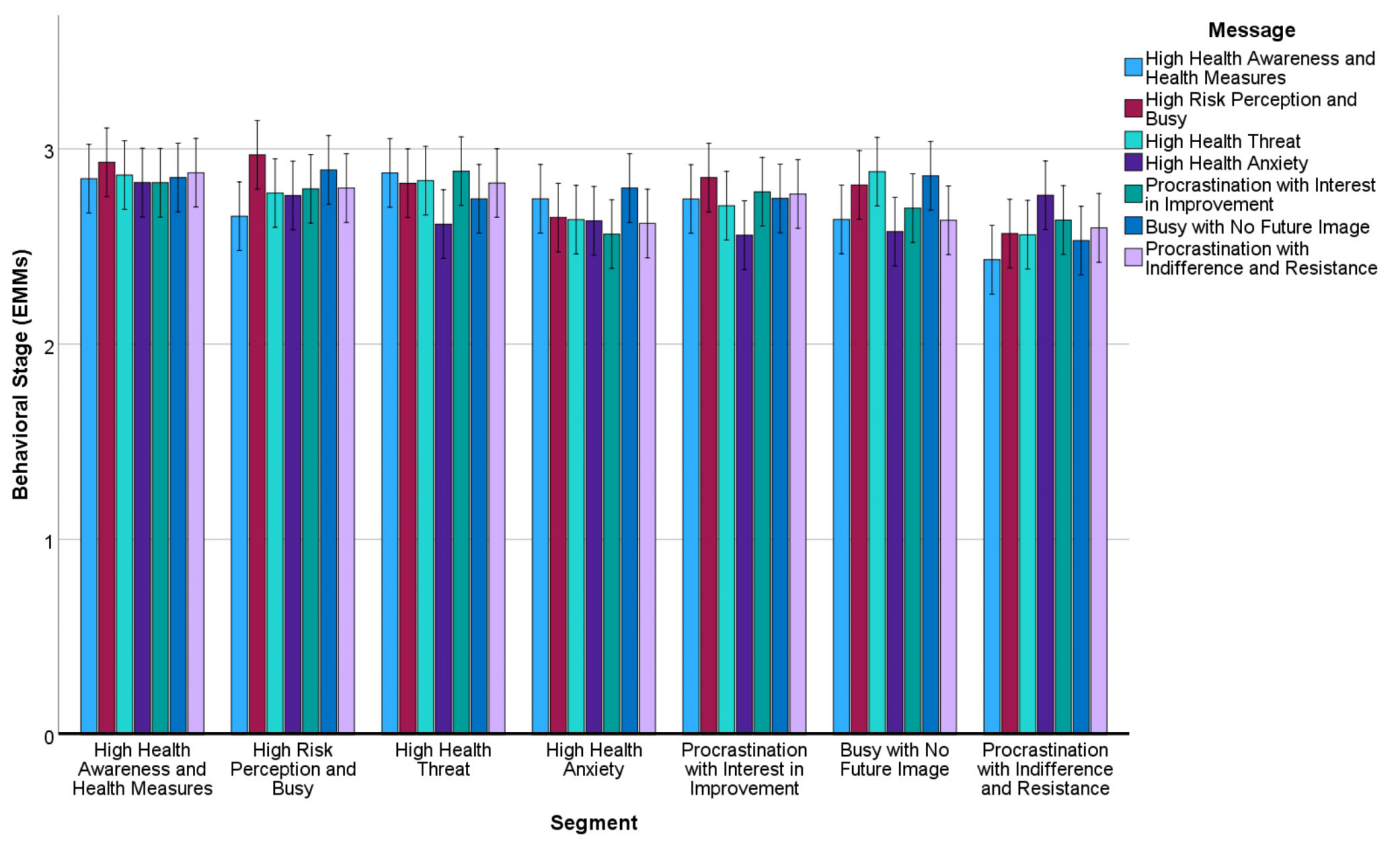
Estimated marginal means of intention to change fitness/sports behaviors after message presentation by segment. Covariates in this model are evaluated at intention to change fitness/sports behaviors (pre-intervention) = 0.000. Error bar: 95% CI.

Factor analysis of 15 health behavior intentions revealed three factors with good reliability: Health Management Behaviors (11 items, α = 0.89, Figure 4), Moderate Physical Activity (2 items, α = 0.76, Figure 5), and Fitness/Sports Behaviors (2 items, α = 0.71, Figure 6) (Table 4).

Significant segment-by-message interactions emerged for Health Management Behaviors (*F* = 3.24, *df* = 36.4850, *p* < 0.01, η*p*^2^ = 0.008) and Moderate Physical Activity (*F* = 2.18, *df* = 36,4850, *p* < 0.05, η*p*^2^ = 0.005). Messages emphasizing immediate, achievable actions showed greatest effectiveness across segments, particularly for the Busy with No Future Image segment (*d* = 0.33, 95% CI: 0.18–0.48).

#### 3.2.4 Temporal framing effects

Messages incorporating 10-year future health projections showed particular effectiveness for segments with high health threat perception and risk awareness (*d* = 0.29, 95% CI: 0.14–0.44, *p* < 0.01). However, segments characterized by strong present bias (Procrastination groups) responded better to messages emphasizing immediate, concrete actions (*d* = 0.24, 95% CI: 0.09–0.39, *p* < 0.05).

## 4 Discussion

This study developed a novel segmentation algorithm for individuals in the precontemplation stage by incorporating behavioral economics concepts and validated targeted messages for each segment. Our findings provide several important theoretical and practical contributions to health behavior change intervention design that extend beyond traditional stage-based approaches.

### 4.1 Novel contributions to understanding precontemplation stage

The development of this segmentation algorithm represents a significant advancement in understanding the heterogeneity within the precontemplation stage. Previous research suggested that this stage might encompass various psychological states beyond simple “resistance to change” (6), but empirical evidence for distinct subgroups was limited. Our analysis revealed seven interpretable segments with unique characteristics, supporting the theoretical proposition that behavioral economic factors create meaningful individual differences in health decision-making.

The primary algorithmic division around future orientation (“Do you have expectations for your future?”) proved particularly significant, separating participants into goal-oriented vs. status quo reference points. This distinction reflects fundamental differences in individuals' ability to conceptualize future health behaviors and aligns with theoretical frameworks in temporal self- regulation (21). These insights have profound implications for health guidance practices, suggesting that traditional goal-setting approaches may not resonate with approximately half of individuals in the precontemplation stage, consistent with recent meta-analytic findings on intervention effectiveness (7).

### 4.2 Segment-specific characteristics and intervention implications

Goal-oriented segments demonstrated active engagement in health behaviors despite relatively low risk perception, suggesting intrinsic motivation rather than fear-based decision-making. This pattern aligns with self-determination theory research showing that autonomous motivation predicts sustained behavior change better than controlled motivation (22). Recent meta-analytic evidence has further consolidated this position, demonstrating that autonomous forms of motivation are consistently associated with health behavior engagement and maintenance across diverse populations and health domains (23, 24). For these segments, interventions should emphasize future gains and potential improvements while maintaining alignment with evidence-based outcomes. Professional guidance through regular health check-ups plays a crucial role in this strategy.

Status quo segments exhibited varying degrees of present bias and resistance to change, consistent with behavioral economics research on temporal discounting (21). The “Procrastination with Interest in Improvement” segment showed receptivity to small, immediate gains, while the “Procrastination with Indifference and Resistance” segment demonstrated stronger present bias. For these groups, interventions focusing on immediate, achievable gains and implementing default options may effectively address present bias patterns (15).

### 4.3 Message validation findings

The validation of targeted messages provided empirical support for the behavioral economics-based segmentation approach. The “High Risk Perception and Busy” and “High Health Threat” segments showed greatest responsiveness to motivational messages, with moderate effect sizes that compare favorably to other health communication interventions. Most notably, the “Procrastination and Improvement Resistance” segment, while showing consistent low responsiveness to comprehensive lifestyle improvement messages, demonstrated increased receptivity to gain-framed messages focusing on specific, concrete actions.

These findings extend behavioral economics theory by demonstrating how reference points and temporal orientation influence message effectiveness in real-world health communication contexts. The differential response patterns across segments support the theoretical proposition that cognitive biases create meaningful individual differences in intervention responsiveness, with practical implications for precision health communication approaches. Our results are consistent with recent evidence showing that individually tailored loss-framed and temporally-framed messages enhance persuasion effectiveness (19), while extending this work by demonstrating the value of comprehensive, multi-dimensional segmentation rather than single-dimension targeting.

### 4.4 Practical implications for health communication

Our findings offer actionable insights for health behavior intervention design across multiple settings. The identification of distinct segments with varying message responsiveness suggests that precision health communication approaches may substantially improve intervention effectiveness compared to one-size-fits-all strategies. Healthcare providers and public health practitioners should consider implementing brief screening tools to identify patient segments and tailor communication accordingly.

The particularly low responsiveness of the “Procrastination and improvement resistance” segment highlights the need for alternative intervention approaches, possibly including environmental modifications or policy-level interventions rather than individual-level messaging. This finding has important implications for resource allocation in health promotion programs, suggesting that some individuals may benefit more from structural interventions than persuasive communication.

An important practical consideration for implementing behavioral economics-based segmentation concerns the feasibility of assessment in real-world health communication contexts. While our simplified 9-item algorithm substantially reduces respondent burden compared to the original 26-variable assessment, even brief measures may encounter resistance in time-constrained clinical or digital health settings where individuals may be reluctant to complete multi-item questionnaires. Recent work on ultra-abridged individual difference scales for digital health personalization suggests that strategic scale shortening—potentially to as few as 1–3 items per construct—may preserve sufficient predictive validity for personalization purposes while substantially improving completion rates and user engagement (25). Future development of our segmentation algorithm should explore whether further item reduction using item response theory or machine learning-based item selection can maintain acceptable classification accuracy while minimizing respondent burden. Additionally, passive behavioral indicators derived from digital health platforms (e.g., app usage patterns, response latencies) could supplement or partially replace self-report measures, enabling less intrusive yet more dynamic segmentation.

### 4.5 Study limitations and future directions

Several limitations warrant consideration in interpreting our findings. Our internet-based recruitment may have introduced selection bias, potentially overrepresenting individuals with higher digital literacy and health consciousness. This could limit generalizability to populations with limited internet access or lower health engagement. The cross-sectional design of Phase 1 prevents causal inferences about relationships between behavioral economic factors and health behaviors.

The classification accuracy of 50.7%, while significantly above chance for a 7-category classification, indicates substantial room for improvement. While this represents a meaningful limitation for practical implementation, several contextual factors help frame this accuracy level (see Supplementary Tables S3, S4 for the complete confusion matrix and segment-specific accuracy). First, the simplified algorithm uses only 9 items compared to the original 26 variables, representing a deliberate trade-off between practical feasibility and classification precision. In real-world health guidance settings, administering a 26-item assessment may not be feasible, whereas a 9-item screener can be integrated into routine clinical workflows. Second, misclassification does not necessarily result in entirely ineffective messaging, as some segments share overlapping characteristics (e.g., Segments 5 and 7 both exhibit procrastination tendencies), meaning that messages designed for adjacent segments may still provide partial benefit compared to generic, non-tailored communication. Nevertheless, improving classification accuracy remains a critical priority for future work. Future research should explore more sophisticated machine learning approaches, including ensemble methods and deep learning techniques, while incorporating objective health measures alongside self-report data.

Furthermore, an important distinction exists between the controlled experimental environment of our message testing and real-world intervention delivery contexts. In our study, participants read approximately 200-word messages within a structured online survey, likely with greater attention and cognitive engagement than would occur in naturalistic settings. In practice, health messages delivered via SMS, smartphone applications, or patient portals compete with numerous other stimuli and may receive only brief, partial attention. Message processing depth, a critical determinant of persuasive effectiveness (26), is likely to be substantially lower in real-world contexts. Future research should therefore investigate not only message content effectiveness but also the impact of delivery modality, message length optimization, timing, and frequency on engagement and processing. Shorter, more concise message formats adapted from the current 200-word versions may be necessary for effective real-world implementation, particularly via mobile platforms where brief, actionable messages can be effective (27).

Our message validation study measured immediate responses rather than sustained behavioral change. The observed effects on intention and stage may not translate to actual behavior modification or long-term health outcomes. Randomized controlled trials with extended follow-up periods are needed to establish intervention effectiveness for objective health behaviors and clinical outcomes.

Cultural factors may limit generalizability beyond the Japanese context. Behavioral economic concepts like present bias and reference points may manifest differently across cultures with varying temporal orientations and collective vs. individualistic values. Cross-cultural validation studies are essential before international implementation.

### 4.6 Future research directions

Future research should prioritize several key directions that build upon the growing literature on personalized behavioral interventions: developing more refined questionnaire items validated through psychometric testing across diverse populations, with particular attention to scale brevity requirements for digital implementation contexts (25); creating sophisticated segmentation algorithms using advanced machine learning and multi-modal data sources, building on recent frameworks for personalized nudging that emphasize individual-level variation in responsiveness to different choice architectures (16, 17); conducting longitudinal field studies with objective behavioral and health outcomes, extending laboratory findings such as those demonstrating the utility of individually tailored loss-framed and temporally-framed messages (18, 19); investigating optimal message delivery frequencies, timing, and channels; exploring cost-effectiveness compared to standard care; and developing implementation guidelines for healthcare systems.

Additionally, research should examine the stability of segment membership over time and identify factors that predict transitions between segments. Understanding these dynamics could inform the development of adaptive intervention systems that adjust messaging strategies based on changing individual characteristics.

### 4.7 Conclusion

This study demonstrates that incorporating behavioral economics concepts into audience segmentation can enhance understanding of the precontemplation stage and improve targeted health communication effectiveness. Our findings suggest that a more nuanced, segment-specific approach to health behavior change interventions may prove more effective than traditional stage-based approaches, particularly for engaging resistant populations.

The identification of seven distinct segments with unique behavioral economic characteristics provides a foundation for developing more personalized health interventions. The differential responsiveness to targeted messages across segments supports the value of precision health communication approaches, while highlighting the challenge of engaging highly resistant individuals who may require alternative intervention strategies.

Future research should focus on refining segmentation algorithms, validating interventions in diverse populations, and demonstrating sustained behavioral change through rigorous randomized controlled trials. The integration of behavioral economics principles into health communication represents a promising direction for addressing the persistent challenge of engaging individuals resistant to health behavior change, with significant implications for improving population health outcomes.

## Data Availability

The datasets generated and analyzed during the current study are not publicly available due to privacy restrictions and ethical approval conditions, but anonymized data may be available from the corresponding author upon reasonable request and with appropriate ethical approval for secondary analysis.

## References

[1] Tsushita K Hosler AS Miura K Ito Y Fukuda T Kitamura A . Rationale and descriptive analysis of specific health guidance: the nationwide lifestyle intervention program targeting metabolic syndrome in Japan. J Atheroscler Thromb. (2018) 25:308–22. doi: 10.5551/jat.42010 29238010 PMC5906184

[2] Nakao YM Miyamoto Y Ueshima K Nakao K Nakai M Nishimura K . Effectiveness of nationwide screening and lifestyle intervention for abdominal obesity and cardiometabolic risks in Japan: the MetS ACTION-J study. PLoS ONE. (2018) 13:e0190862. doi: 10.1371/journal.pone.0190862 29315322 PMC5760033

[3] Prochaska JO Velicer WF. The transtheoretical model of health behavior change. Am J Health Promot. (1997) 12:38–48. doi: 10.4278/0890-1171-12.1.38 10170434

[4] Adams J White M. Why don't stage-based activity promotion interventions work? Health Educ. Res. (2005) 20:237–43. doi: 10.1093/her/cyg105 15253998

[5] Bridle C Riemsma RP Pattenden J Sowden AJ Mather L Watt IS . Systematic review of the effectiveness of health behavior interventions based on the transtheoretical model. Psychol Health. (2005) 20:283–301. doi: 10.1080/08870440512331333997

[6] Hall PA Fong GT. Temporal self-regulation theory: a model for individual health behavior. Health Psychol Rev. (2007) 1:6–52. doi: 10.1080/17437190701492437

[7] Noar SM Benac CN Harris MS. Does tailoring matter? Meta-analytic review of tailored print health behavior change interventions. Psychol. Bull. (2007) 133:673–93. doi: 10.1037/0033-2909.133.4.673 17592961

[8] Joyal-Desmarais K Scharmer AK Madzelan MK See JV Rothman AJ Snyder M. Appealing to motivation to change attitudes, intentions, and behavior: a systematic review and meta-analysis of 702 experimental tests of the effects of motivational message matching on persuasion. Psychol Bull. (2022) 148:465–517. doi: 10.1037/bul0000377

[9] Kreuter MW Wray RJ. Tailored and targeted health communication: strategies for enhancing information relevance. Am J Health Behav. (2003) 27:S227–32. doi: 10.5993/AJHB.27.1.s3.6 14672383

[10] Loewenstein G Brennan T Volpp KG. Asymmetric paternalism to improve health behaviors. JAMA. (2007) 298:2415–7. doi: 10.1001/jama.298.20.2415 18042920

[11] Thaler RH Sunstein CR. Nudge: Improving Decisions about Health, Wealth, and Happiness. New Haven: Yale University Press (2008).

[12] O'Donoghue T Rabin M. Doing it now or later. Am Econ Rev. (1999) 89:103–24. doi: 10.1257/aer.89.1.103

[13] O'Donoghue T Rabin M. The economics of immediate gratification. J Behav Decis Making. (2000) 13:233–50. doi: 10.1002/(SICI)1099-0771(200004/06)13:23.0.CO;2-U

[14] Rothman AJ Baldwin AS Hertel AW Fuglestad PT. Self-regulation and behavior change: disentangling behavioral initiation and behavioral maintenance. In:Vohs KD and Baumeister RF, editors. Handbook of Self-Regulation: Research, Theory, and Applications, 2nd Edn. New York: Guilford Press (2011). p. 106–22.

[15] Rogers T Milkman KL Volpp KG. Commitment devices: using initiatives to change behavior. JAMA. (2014) 311:2065–6. doi: 10.1001/jama.2014.3485 24777472

[16] Mills S. Personalized nudging. Behav Public Policy. (2022) 6:150–9. doi: 10.1017/bpp.2020.7

[17] Peer E Mills S. From one, many: how can nudges be personalized? Behav. Sci. Policy. (2026). doi: 10.1177/23794607251403327. [Epub ahead of print].

[18] de Vries R Bol N van der Laan N. “Just-in-time” but a bit delayed: Personalizing digital nudges for healthier online food choices. Appetite. (2025) 206:107852. doi: 10.1016/j.appet.2025.107852 39778812

[19] Tripp HL Strickland JC Mercincavage M Audrain-McGovern J Donny EC Strasser AA. Tailored cigarette warning messages: how individualized loss aversion and delay discounting rates can influence perceived message effectiveness. Int J Environ Res Public Health. (2021) 18:10492. doi: 10.3390/ijerph181910492 34639792 PMC8507605

[20] Ministry Ministry of Health, Labour Labour and Welfare of Japan. Report on the development of effective health guidance methods based on behavioral economics for lifestyle-related disease prevention. Report No.: 202109046A. Tokyo: Ministry of Health, Labour and Welfare of Japan (2021). [In Japanese]

[21] Story GW Vlaev I Seymour B Darzi A Dolan RJ. Does temporal discounting explain unhealthy behavior? A systematic review and reinforcement learning perspective. Front Behav Neurosci. (2014) 8:76. doi: 10.3389/fnbeh.2014.00076 24659960 PMC3950931

[22] Ryan RM Deci EL. Self-determination theory and the facilitation of intrinsic motivation, social development, and well-being. Am Psychol. (2000) 55:68–78. doi: 10.1037//0003-066X.55.1.68 11392867

[23] Ntoumanis N Ng JYY Prestwich A Quested E Hancox JE Thogersen-Ntoumani C . A meta-analysis of self-determination theory-informed intervention studies in the health domain: effects on motivation, health behavior, physical, and psychological health. Health Psychol Rev. (2021) 15:214–44. doi: 10.1080/17437199.2020.1718529 31983293

[24] Sheeran P Wright CE Avishai A Villegas ME Lindemans JW Klein WMP . Self-determination theory interventions for health behaviour change: meta-analysis and meta-analytic structural equation modelling of randomized controlled trials. J Consult Clin Psychol. (2020) 88:726–37. doi: 10.1037/ccp0000501 32437175

[25] Yeung SK Tong ACY Zhao H Mak WWS. Using ultra abridged individual difference scales for personalization in digital mental health to improve uptake, engagement, and experiences: a three-tiered decision framework for scale shortening. PsyArXiv [Preprint]. (2025). doi: 10.31234/osf.io/d5a48_v2 PMC13247296 42258804

[26] Petty RE Cacioppo JT. The elaboration likelihood model of persuasion. Adv Exp Soc Psychol. (1986) 19:123–205. doi: 10.1016/S0065-2601(08)60214-2

[27] Head KJ Noar SM Iannarino NT Grant Harrington N. Efficacy of text messaging-based interventions for health promotion: a meta-analysis. Soc Sci Med. (2013) 97:41–8. doi: 10.1016/j.socscimed.2013.08.003 24161087

