# Supplementary material for: Development of a new segmentation algorithm for “precontemplation” stage in lifestyle change among high-risk populations for lifestyle-related diseases
Source: Front Public Health. 2026 Apr 10;14:1769487. doi: 10.3389/fpubh.2026.1769487 (PMC13106352; doi:10.3389/fpubh.2026.1769487)
Supplement: Supplementary file 1 [file Data_Sheet_1.docx]

**Supplementary Material**

**Supplementary Table S1. Characteristics of Study Participants by Cluster Groups (Phase 1, n = 1,125)**

| **Variable** | **Category** | **Seg 1 (n=210)** | | **Seg 2 (n=135)** | | **Seg 3 (n=143)** | | **Seg 4 (n=51)** | | **Seg 5 (n=133)** | | **Seg 6 (n=216)** | | **Seg 7 (n=237)** | | **χ²** | **p** |
| --- | --- | --- | --- | --- | --- | --- | --- | --- | --- | --- | --- | --- | --- | --- | --- | --- | --- |
|  |  | **N** | **%** | **N** | **%** | **N** | **%** | **N** | **%** | **N** | **%** | **N** | **%** | **N** | **%** |  |  |
| **BMI** | High | 8 | 3.8% | 13 | 9.6% | 17 | 11.9% | 14 | 27.5% | 28 | 21.1% | 24 | 11.1% | 27 | 11.4% | 36.97 | < .001 |
| **Specific Health Guidance** | Received | 120 | 57.1% | 97 | 71.9% | 70 | 49.0% | 24 | 47.1% | 78 | 58.6% | 126 | 58.3% | 126 | 53.2% | 22.22 | .04 |
| **Abnormal Findings in Checkups** | Multiple times | 82 | 39.0% | 63 | 46.7% | 76 | 53.1% | 18 | 35.3% | 76 | 57.1% | 96 | 44.4% | 136 | 57.4% | 63.66 | < .001 |
|  | Once | 71 | 33.8% | 44 | 32.6% | 36 | 25.2% | 12 | 23.5% | 29 | 21.8% | 58 | 26.9% | 74 | 31.2% |  |  |
| **Intention to Improve Lifestyle** | No intention | 62 | 29.5% | 22 | 16.3% | 47 | 32.9% | 38 | 74.5% | 37 | 27.8% | 77 | 35.6% | 28 | 11.8% | 162.18 | < .001 |
|  | Intends to improve | 39 | 18.6% | 42 | 31.1% | 39 | 27.3% | 5 | 9.8% | 45 | 33.8% | 66 | 30.6% | 68 | 28.7% |  |  |
|  | Has started gradually | 20 | 9.5% | 19 | 14.1% | 25 | 17.5% | 3 | 5.9% | 23 | 17.3% | 36 | 16.7% | 41 | 17.3% |  |  |
|  | Already working on | 37 | 17.6% | 29 | 21.5% | 12 | 8.4% | 0 | 0.0% | 20 | 15.0% | 23 | 10.6% | 50 | 21.1% |  |  |
|  | Maintaining (6+ mo) | 52 | 24.8% | 23 | 17.0% | 20 | 14.0% | 5 | 9.8% | 8 | 6.0% | 14 | 6.5% | 50 | 21.1% |  |  |

*Note: Seg 1 = High Health Awareness and Practices; Seg 2 = High Risk Perception and Busy; Seg 3 = Procrastination and Improvement Interest; Seg 4 = Procrastination and Improvement Resistance; Seg 5 = High Health Anxiety; Seg 6 = Busy and No Future Image; Seg 7 = High Health Threat.*

**Supplementary Table S2. Questionnaire Items Used in Segmentation Analysis**

| **Category** | **Question Content** | **Used in Analysis** |
| --- | --- | --- |
| **Self-determining ability** | Do you find making decisions difficult? | 1 |
|  | Do you take a long time to make decisions? | 0 |
|  | Do you try to avoid making decisions? | 0 |
|  | Do you prefer others to make decisions for you? | 0 |
|  | Do you often change your decisions after making them? | 0 |
| **Responding to High Goals** | When trying to do something, do you set high goals and try to achieve them all at once, or do you prefer to start with what you can do little by little? | 1 |
| **View on Perfect Achievement** | When trying to do something, do you aim for perfect achievement of goals, or are you satisfied with partial achievement? | 1 |
| **Degree of Goal Achievement** | Do you feel you have achieved the things you wanted to do and the goals you set in your life so far? | 1 |
| **Life Satisfaction Level** | Are you satisfied with your life? | 1 |
| **Anxiety about Future Life** | Do you feel anxious about your future life? | 1 |
| **Expectations for Own Future** | Do you have expectations for your future? | 1 |
| **Desire for Long Life** | Do you wish to have a long remaining life? | 1 |
| **Possibility of Lifestyle Improvement** | Do you think you could improve your lifestyle habits if you really tried? | 1 |
| **Current Health is Sufficient** | I think I am sufficiently healthy now | 1 |
| **Feel Will Get Serious Illness** | I feel I might develop a serious illness if I continue my current lifestyle | 1 |
| **Don't Mind Spending on Health** | I don't mind spending money and time on health | 1 |
| **Don't Care about Health** | I don't pay attention to my health status because my body is only mine | 1 |
| **Checking Health Status** | I am conscious of and check my health status daily | 1 |
| **Catchphrase is 'Busy'** | 'I'm busy' has become my catchphrase | 1 |
| **Decision Making (Deliberation)** | Are you an intuitive decision-maker or do you prefer to think things through carefully? | 1 |
| **Successful Experience** | Have you successfully quit smoking, reduced alcohol, or lost weight? | 1 |
| **Success in Non-Health Activities** | Have you taken up hobbies or studied for qualifications? | 1 |
| **Failure Experience** | Have you failed in attempts to quit smoking, reduce alcohol, or lose weight? | 1 |
| **Major Disease Risk Recognition** | Are you worried about developing brain disease? | 1 |
|  | Are you worried about developing cancer? | 0 |
|  | Are you worried about developing heart disease? | 0 |
|  | Are you worried about developing dementia? | 0 |
| **Mental Health Loss Risk** | Are you worried about developing a depressed lifestyle? | 1 |
|  | Are you worried about losing your sense of purpose? | 0 |
| **Lifestyle Disease Risk** | Are you worried about becoming obese? | 1 |
|  | Are you worried about developing hypertension? | 0 |
| **Disease Fear** | Do you consider heart disease frightening? | 1 |
| **Lifestyle Improvement Self-Efficacy** | How much do you think you could reduce future disease risk by changing your lifestyle? | 1 |
| **Present Bias (Summer Homework)** | When you were a child, when did you usually do your holiday homework? | 1 |
| **Risk Avoidance Level (Umbrella)** | At what percentage chance of rain do you take an umbrella? | 1 |

*Note: 1 = Item used in final segmentation analysis; 0 = Item not used (excluded based on factor analysis or redundancy).*

**Supplementary Table S3. Confusion Matrix for CHAID Classification Algorithm**

This table presents the confusion matrix comparing the original 26-variable k-means clustering assignments (rows) with the simplified 9-item CHAID algorithm predictions (columns). Overall classification accuracy was 50.7% (570/1,125), with Cohen's κ = 0.42 (95% CI: 0.39–0.45).

| **Original Cluster** | **CHAID Predicted Segment** | | | | | | | **Total** |
| --- | --- | --- | --- | --- | --- | --- | --- | --- |
|  | **1** | **2** | **3** | **4** | **5** | **6** | **7** |  |
| **1** | **119** | 16 | 37 | 3 | 2 | 23 | 10 | **210** |
| **2** | 25 | **53** | 9 | 0 | 4 | 24 | 20 | **135** |
| **3** | 2 | 3 | **62** | 11 | 37 | 16 | 12 | **143** |
| **4** | 3 | 0 | 2 | **33** | 3 | 10 | 0 | **51** |
| **5** | 0 | 0 | 10 | 3 | **113** | 5 | 2 | **133** |
| **6** | 19 | 20 | 23 | 6 | 29 | **108** | 11 | **216** |
| **7** | 8 | 9 | 22 | 0 | 67 | 49 | **82** | **237** |
| **Total** | **176** | **101** | **165** | **56** | **255** | **235** | **137** | **1,125** |

*Note: Green shading indicates correct classifications (diagonal). Segment labels: 1 = High Health Awareness and Practices; 2 = High Risk Perception and Busy; 3 = Procrastination and Improvement Interest; 4 = Procrastination and Improvement Resistance; 5 = High Health Anxiety; 6 = Busy and No Future Image; 7 = High Health Threat. The largest off-diagonal value (67) occurred between Segments 7 and 5, both characterized by elevated health concerns.*

**Supplementary Table S4. Classification Accuracy by Segment**

| **Segment** | **Correct** | **Total** | **Accuracy** |
| --- | --- | --- | --- |
| 1. High Health Awareness and Practices | 119 | 210 | 56.7% |
| 2. High Risk Perception and Busy | 53 | 135 | 39.3% |
| 3. Procrastination and Improvement Interest | 62 | 143 | 43.4% |
| 4. Procrastination and Improvement Resistance | 33 | 51 | 64.7% |
| 5. High Health Anxiety | 113 | 133 | 85.0% |
| 6. Busy and No Future Image | 108 | 216 | 50.0% |
| 7. High Health Threat | 82 | 237 | 34.6% |
| **Overall** | **570** | **1,125** | **50.7%** |

*Note: Classification accuracy varied substantially across segments, with Segment 5 (High Health Anxiety) showing highest accuracy (85.0%) and Segment 7 (High Health Threat) showing lowest accuracy (34.6%). This variation reflects differences in segment distinctiveness within the CHAID decision tree structure.*
